# Insights from Genomics into Bacterial Pathogen Populations

**DOI:** 10.1371/journal.ppat.1002874

**Published:** 2012-09-06

**Authors:** Daniel J. Wilson

**Affiliations:** 1 Wellcome Trust Centre for Human Genetics, University of Oxford, Oxford, United Kingdom; 2 Nuffield Department of Clinical Medicine, Experimental Medicine Division, University of Oxford, Oxford, United Kingdom; International Centre for Genetic Engineering and Biotechnology, India

## Abstract

Bacterial pathogens impose a heavy burden of disease on human populations worldwide. The gravest threats are posed by highly virulent respiratory pathogens, enteric pathogens, and HIV-associated infections. Tuberculosis alone is responsible for the deaths of 1.5 million people annually. Treatment options for bacterial pathogens are being steadily eroded by the evolution and spread of drug resistance. However, population-level whole genome sequencing offers new hope in the fight against pathogenic bacteria. By providing insights into bacterial evolution and disease etiology, these approaches pave the way for novel interventions and therapeutic targets. Sequencing populations of bacteria across the whole genome provides unprecedented resolution to investigate (i) within-host evolution, (ii) transmission history, and (iii) population structure. Moreover, advances in rapid benchtop sequencing herald a new era of real-time genomics in which sequencing and analysis can be deployed within hours in response to rapidly changing public health emergencies. The purpose of this review is to highlight the transformative effect of population genomics on bacteriology, and to consider the prospects for answering abiding questions such as why bacteria cause disease.

## Introduction

Bacteria are the most abundant group of organisms, and a major source of human disease and mortality. Bacterial cells account for most of the earth's biomass [1], and the 100 trillion microbial residents of the human body outnumber human cells 10 to 1 [2]. Bacteria that cause pneumonia, diarrhea, and tuberculosis are leading causes of death worldwide [3], [4]. In countries with a low overall burden of infectious disease such as the United States, bacteria are nevertheless responsible for more than 60% of the deaths attributable to communicable disease, with hospital-associated infections, HIV-associated infections, and tuberculosis most prominent (Table 1).

**Table 1:** Major bacterial causes of death: World and United States.

Cause of Death	Total Deaths (Thousands)	% Communicable Disease Deaths	Key Bacterial Species
Global (2008 estimates) [3], [83]			
Lower respiratory infections	3,742	30.6	*Streptococcus pneumoniae*, *Haemophilus influenzae*
Tuberculosis	1,833	15.0	*Mycobacterium tuberculosis*
Directly attributable	1,250	10.2	
HIV-associated^a^	583	4.8	
Diarrhoeal disease	1,687	13.8	*Vibrio cholerae*, *Escherichia coli*, *Salmonella typhi*
Meningitis	270	2.2	*Neisseria meningitidis*
Pertussis	194	1.6	*Bordetella pertussis*
Tetanus	128	1.0	*Clostridium tetani*
Syphilis	81	0.7	*Treponema pallidum*
Upper respiratory infections	69	0.6	*Streptococcus pyogenes*
Chlamydia	7	0.1	*Chlamydia trachomatis*
Other communicable disease^b^	4,231	34.5	
United States of America (1999–2007) [84]			
Sepsis^b^	280.3	48.17	
Clostridium difficile infection	30.2	5.19	*Clostridium difficile*
Staphylococcal infection	16.6	2.86	*Staphylococcus aureus*
HIV-associated^b^	9.7	1.66	
Tuberculosis	8.8	1.50	*Mycobacterium tuberculosis*
Directly attributable	7.4	1.26	
HIV-associated	1.4	0.24	
Streptococcal infection	6.4	1.09	*Streptococcus pneumoniae*
Meningococcal disease	1.4	0.24	*Neisseria meningitidis*
Legionnaires' disease	0.7	0.12	*Legionella pneumophila*
Other bacterial disease^b^	17.6	4.57	
Other communicable disease^b^	210.1	36.1	

The total number of deaths attributable to communicable diseases is shown for the world (2008 estimates) and United States (1999–2007), with key bacterial species highlighted. At the global level, the WHO classifications for causes of death are broad and usually encompass multiple etiological agents, not only bacterial species. The United States and some other countries classify deaths based on detailed ICD-10 four-digit codes that frequently specify the bacterial species responsible.

a Estimated from the total number of HIV deaths assuming 26% are associated with tuberculosis [85].

b Excluding other causes of death mentioned explicitly.

Since the introduction of the earliest antibiotics, bacteria have evolved resistance [5]. Treatment options continue to be eroded by the spread of antibiotic resistance [6], not only in countries with advanced health care infrastructure, but globally [7], [8]. However, advances in DNA sequencing capacity offer hope in the fight against pathogenic bacteria because the ability to sequence populations of bacterial genomes is illuminating our understanding of bacterial evolution and virulence. Ultimately these insights will underpin translational research into improved medical practice, drug and vaccine targets, and public health policy.

**Figure 1:**
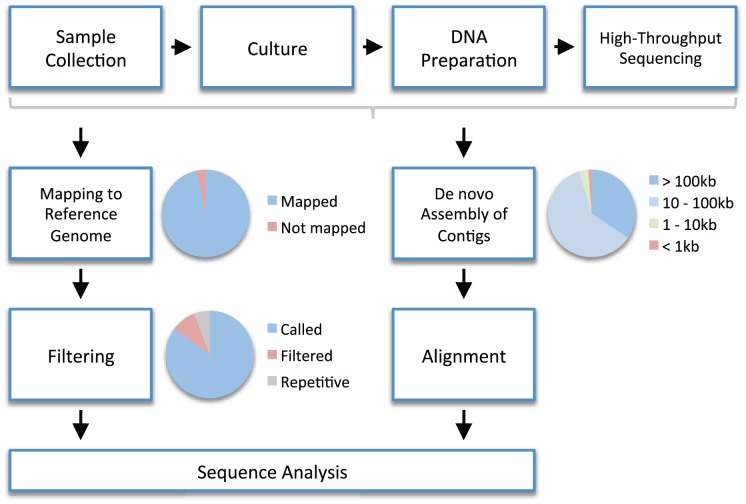
An example workflow for high-throughput whole genome sequencing in bacteria. *Sample collection*. A biological sample (e.g., blood) is collected. *Culture*. Bacterial colonies are isolated from the sample by culturing on appropriate media. *DNA Preparation*. DNA is extracted from the colonies and a DNA library is prepared ready for sequencing. *High-Throughput Sequencing*. Millions of short sequence reads are yielded, typically several hundred nucleotides long or less. To reconstruct the genome, one of two approaches is generally adopted. *Mapping to Reference Genome*. In reference-based mapping, the short sequences are mapped (i.e., aligned) to a reference genome using an algorithm (e.g., [73], [74]). Preferably the reference genome is high quality, complete, and closely related. The pie chart illustrates that not all reads necessarily map to the reference genome (e.g., because of novel regions not present in the reference). *Filtering*. Short reads cannot be mapped reliably to repetitive regions of the reference genome, so these are identified and filtered out. Sites that are problematic for other reasons (e.g., because too few reads have mapped or because the consensus nucleotide is ambiguous) are also filtered out. The pie chart illustrates that some portion of the reference genome does not get called due to filtering. In the mapped genome, these positions will receive an ambiguity code (i.e., N rather than A, C, G, or T). *De novo Assembly of Contigs*. An alternative to mapping is de novo assembly, in which no reference genome is used. An algorithm (e.g., [75], [76]) is used to assemble short reads into longer sequences known as contigs. The number and length of contigs will depend on general factors such as the length of sequence reads and the total amount of DNA sequence produced, as well as local factors such as the presence of repetitive regions. The pie chart shows an example of the proportion of all reads that assemble into contigs of a given length. *Alignment*. For further analysis, it is necessary to align local regions (e.g., genes) or whole genomes using appropriate algorithms (e.g., [77]–[79]). There is a trade-off in computational terms between the length of region and the number of sequences that can be aligned. *Sequence Analysis*. The two approaches produce sequence alignments that represent pairwise alignments against a reference (mapping) or multiple alignments one to another (de novo assembly). These alignments can be analyzed directly, or processed further to detect variants such as single nucleotide polymorphisms, insertions, and deletions. The pie charts are meant to be illustrative only, and were produced from data in [27].

High-throughput whole genome sequencing (Figure 1) represents a genuine step change for the study of bacterial populations because current approaches are based on the analysis of gene fragments amounting to just a few thousandths the total length of the genome [9], [10]. Population genomics offers unprecedented sensitivity for the detection of rare genetic variants, vastly improved resolution for population studies, and direct sequencing of functionally relevant loci. As a result, it is driving new understanding of within-host evolution, transmission, and population structure. Moreover, the advent of rapid benchtop sequencing is changing the way that microbiology is conducted, signaling a new era of real-time genomics and disseminated collaborative analysis in response to rapidly changing public health emergencies.

## Within-Host Evolution

Successful colonization of a host is essential to the lifecycle of the pathogen, and the dynamics of the host-pathogen interaction determine the outcome of the interaction, including the severity of disease. DNA/RNA sequencing has greatly advanced the understanding of viral dynamics during infection [11], [12], including the ability to predict disease progression [13], [14], but progress in bacteria has lagged behind, owing to much larger genomes and sparser genetic variation [15]. However, whole genome sequencing in populations of bacteria colonizing individual hosts is shedding new light on the host-pathogen interaction, and the dynamics of bacterial evolution within the host.

At the whole genome scale, genetic variation has been discovered in singly infected hosts colonized by species as disparate as *Mycobacterium tuberculosis* [16], *Salmonella enterica* [17], and *Staphylococcus aureus* [18]. The absolute number of variable sites detected in singly infected hosts is small, frequently fewer than 10 single nucleotide polymorphisms (SNPs), although this varies by species and depends on the number of genomes sequenced and the time elapsed between sampling. Other forms of genetic variation observable at the species level [19] have also been detected, including short insertions and deletions (indels), and variation in the presence or absence of mobile elements such as prophages [16]–[18].

The real-time mutation rate is a key factor in determining the potential for bacterial pathogens to adapt to the host immune system or drug intervention. Traditional estimates of bacterial substitution rates over geological timescales predict fewer than 0.01 mutations per megabase (Mb) per year [20], [21]. Yet laboratory estimates and limited sequencing of longitudinal samples suggest rates 100 or 1,000 times faster [22]–[26]. These estimates have been put to the test by whole genome sequencing, yielding within-host mutation rates ranging from 0.1/Mb/year in *Mycobacterium tuberculosis* [16] through 2.7/Mb/year in *Staphylococcus aureus* [27] to 19/Mb/year in *Helicobacter pylori* [28]. This supports the conclusion that short-term substitution rates in bacteria are several orders of magnitude faster than long-term rates [23], [26], a finding that may be explained by the delayed action of purifying selection [29], [30]. In other words, over longer evolutionary periods the substitution rate depends on selection as well as mutation. It also demonstrates the potential for bacteria to adapt within the host. For example, the discovery that the genome-wide mutation rate in latent tuberculosis infection is similar to that in active disease may explain reports that found treating even latent infections with the antibiotic isoniazid was a risk factor for the emergence of isoniazid resistance [16].

Many bacterial pathogens are common constituents of the body's natural flora [31]. Evolution during colonization may trigger a transition from healthy carriage to invasive disease. For example, 27% of adults carry *Staphylococcus aureus* asymptomatically in the nose [32], a known risk factor for disease [33]. In a study of one long-term carrier who developed a bloodstream infection, the genomes of invasive bacteria were found to possess an excess of mutations that truncated proteins, including a transcriptional regulator implicated in pathogenicity [27], [34]. Although further work would be needed to establish causality, this demonstrates the potential for loss-of-function mutations to induce radical functional change during colonization.

Unusual patterns of mutation in the genome during colonization may signal adaptive change and reveal mechanisms of virulence or immune evasion. A study of a 16-year outbreak of chronic *Burkholderia dolosa* infection among cystic fibrosis patients revealed evidence for parallel adaptive evolution [35]. Seventeen genes accrued three or more mutations across the 14 patients, of which a significant excess altered the encoded protein. Some of these mutations affected important phenotypes, including oxygen-dependent gene regulation—which may be pertinent to lung infection—antibiotic resistance, and outer membrane synthesis. Mutations not previously implicated in pathogenesis present novel therapeutic targets.

In hosts colonized multiple times by distinct genotypes, whole genome sequencing affords an opportunity to investigate recombination in vivo. Horizontal gene transfer, also known as recombination, is a fundamental process that generates diversity and facilitates the spread of advantageous genes [36], [37]. A longitudinal study of the highly promiscuous gut pathogen *Helicobacter pylori* identified recombination events from the clustering of SNP differences introduced by the import of DNA from one strain to another [28]. Surprisingly, multiple fragments of around 400 bases appeared to be simultaneously imported in a span up to 20 kilobases long. This pattern of integration was implied by the results of a similar study in *Streptococcus pneumoniae* [38], demonstrating the power of whole genome sequencing to illuminate molecular mechanisms.

## Detection of Transmission Events

Whole genome sequencing offers unprecedented resolution to distinguish degrees of relatedness among bacterial isolates, and this is a powerful tool for microbial forensics [39]. Genome sequencing complements existing epidemiological tools by providing a means to reconstruct recent chains of transmission, identify sequential acquisition of strains by persistent carriers, and identify cryptic outbreaks that might otherwise go unnoticed.

The superiority of genomics over traditional approaches to molecular epidemiology was demonstrated in a study of *Staphylococcus aureus* ST-239 [40], a widely disseminated multi-drug resistant clonal lineage dominant in much of Asia. Traditional typing methods offer little discriminatory power for subtyping ST-239, but 5,842 SNPs were discovered by whole genome sequencing, revealing detailed geographical structure within the lineage. Against this backdrop of geographical differentiation, examples of recent intercontinental transmission were evident from the clustering of two isolates from England and Denmark among the Thai group. Moreover, a cluster of five isolates sampled over 11 weeks from adjacent blocks of a Thai hospital differed by just 14 SNPs, providing evidence of recent hospital transmission.

Population genomics offers complementary tools to routine outbreak investigation. Following the discovery of two index cases, an outbreak of *Mycobacterium tuberculosis* was uncovered in British Columbia using contact tracing and social network questionnaires [41]. The transitivity of the social network and inability to distinguish isolates by traditional genotyping prevented identification of the source. Whole genome sequencing revealed two distinct lineages, ruling out transmission between social contacts infected with discordant types. Further epidemiological investigation intimated a complex scenario in which an increase in crack cocaine usage triggered simultaneous outbreaks that were sustained by key members of a high-risk social network.

**Figure 2:**
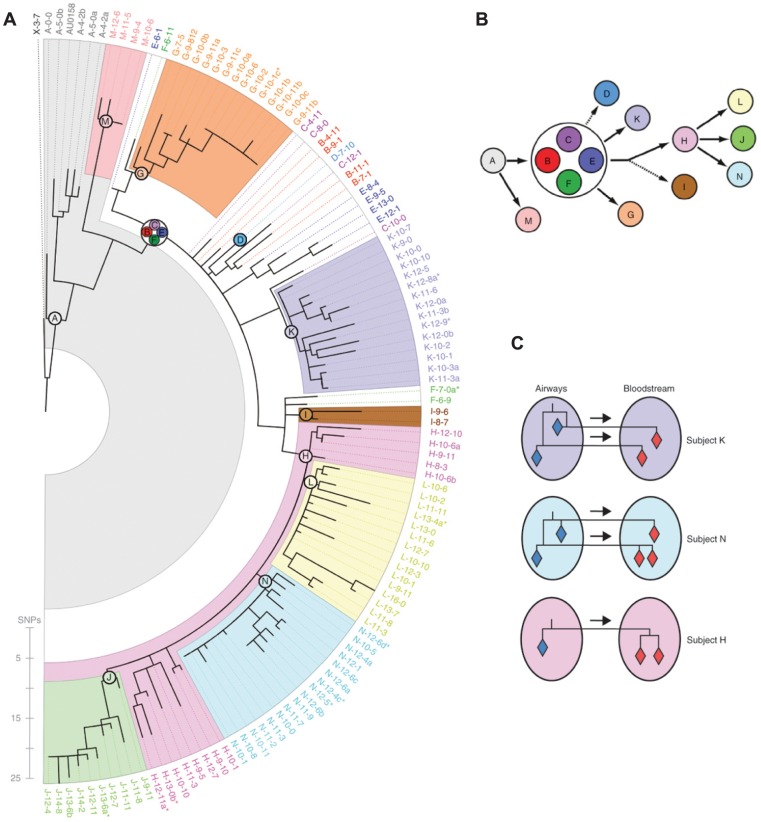
Whole genome sequencing reveals within-host evolution and recent transmission between patients. Lieberman, Michel, and colleagues [35] sequenced the genomes of 112 isolates of *Burkholderia dolosa* from 14 cystic fibrosis patients involved in an outbreak in Boston, Massachusetts in the 1990s. (A) The maximum likelihood tree relating the bacterial genomes, color-coded by patient, is broadly consistent with a single founding infection for each patient. (B) The date of sampling and the chronological accumulation of mutations implied a network of transmission events. (C) Interesting patterns emerged when comparing bacteria isolated from different sites in the same patient. For two patients (subjects K and N), multiple genotypes appeared to have been transmitted from the airways to the bloodstream during septicemia, either concurrently or over the course of the infection. By contrast, a single genotype appeared to have been transmitted from the airways to the bloodstream in subject H. Reproduced from [35] appearing in *Nature Genetics* (Volume 43, 2011).

In some cases, the direction of transmission may be discernible from patterns of relatedness and associated epidemiological information. In their study of chronic *Burkholderia dolosa* infection among cystic fibrosis patients [35], the authors used the chronological accumulation of mutations to discriminate donors from recipients in the transmission network. By the same method, they were even able to infer repeated transmission from the airways to the bloodstream within patients (Figure 2). In a persistent *Escherichia coli* infection of members of a household over three years [42], whole genome sequencing revealed at least six transmission events between family members including at least two zoonotic transmissions to the family dog.

Multiple transmission events resulting in serial acquisition by a single host can be distinguished from persistent or relapsing infection using whole genome sequencing. This is useful in infections such as invasive nontyphoidal *Salmonella*, a common cause of severe and recurring bloodstream infections among HIV-infected adults in Africa. A study of invasive nontyphoidal *Salmonella* in 14 Malawian patients discriminated recrudescent (i.e., relapsing) infection from multiple infection on the basis of relatedness inferred from genome-wide SNP differences [17]. Recrudescence accounted for 78% of recurring infections, although recrudescence and multiple infection in the same patient was not uncommon.

## Historical Patterns of Transmission

In addition to revealing fine-grained genetic structure that is informative about recent transmission, genomics offers unrivalled precision for reconstructing historical patterns of spread. With comprehensive sampling, we can identify the geographical and temporal origin of pandemics and the dominant transmission routes responsible for global dissemination. For example, a study of *Yersinia pestis* used genome sequencing to assist in the discovery of 933 SNPs subsequently typed in 286 global isolates [43]. Based on the diversity and juxtaposition of isolates close to the root of the tree, the authors concluded that the origin of plague more than 2,600 years ago occurred in or near China.

Understanding the circumstances under which epidemics emerge and take hold may help to manage contemporary threats and prevent future outbreaks. The history of the seventh and current pandemic of *Vibrio cholerae* was pieced together using population genomics [44]. An analysis of global isolates revealed three partially overlapping waves of pandemic cholera originating in the Bay of Bengal during the 1950s and leading to a succession of geographically restricted epidemics. Each wave was characterized by a particular armory of genetic elements including distinct forms of the cholera toxin and, from the second wave onwards, SXT/R391 integrative and conjugative elements that confer antibiotic resistance.

**Figure 3:**
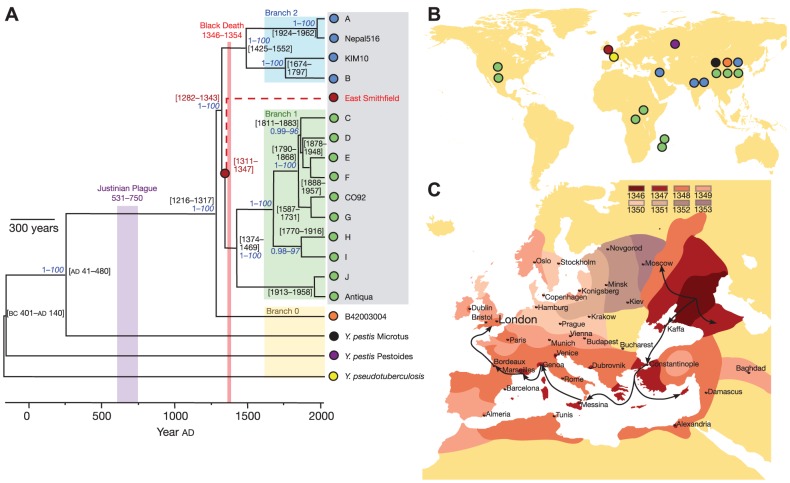
Patterns of historical transmission reconstructed by whole genome sequencing. Bos, Schuenemann, and colleagues [47] combined ancient DNA techniques with whole genome sequencing to reconstruct a draft genome of *Yersinia pestis*, the bacterium responsible for the Black Death, from five teeth recovered from a 660-year-old burial ground. (A) Genealogical reconstruction reveals that the bacteria responsible for the Black Death are positioned ancestral to modern Branch 1 *Yersinia pestis*, close to the most recent common ancestor of all modern *Yersinia pestis* pathogenic to humans. No derived mutations were observed in the ancient genome, suggesting that modern Branch 1 bacteria are essentially equivalent, and that differences in modern and 14^th^ century epidemiology probably do not result from genetic changes in the bacteria. (B) Geographical origin of the bacterial isolates. (C) Inferred geographical spread of the Black Death through Europe [80]. Reproduced from [47] appearing in *Nature* (Volume 478, 2011).

Sequencing ancient bacteria is a particularly powerful tool for investigating historical transmission. To reconstruct the history of leprosy, SNPs discovered by whole genome sequencing were typed in over 400 isolates of *Mycobacterium leprae*, including bacteria isolated from skeletal remains recovered from leprosy graveyards in and around Europe [45]. The paleomicrobiological samples resembled modern European isolates, supporting a model in which leprosy arose in East Africa before dispersing east and west by traders along the Silk Road [46]. The provenance of *Yersinia pestis* was investigated by sequencing bacteria isolated from teeth disinterred from the East Smithfield burial ground for Black Death victims in London [47]. The reconstructed genome closely resembled the most recent common ancestor of modern plague in humans, suggesting that the Black Death was the main historical event antecedent to contemporary plague worldwide (Figure 3).

Zoonosis is a major source of emerging infectious disease, with wildlife the most frequent origin [48]. In the United States, leprosy is rare and most infected individuals have a history of foreign residence. Yet a third of cases in Texas and Louisiana had no such explanation [49]. Genome sequencing and SNP typing revealed that a distinctive strain of leprosy was present in 33 wild armadillos and 26 of 29 unexplained human cases, strongly suggesting zoonotic transmission of *Mycobacterium leprae* from wild armadillos.

## Population Structure, Carriage, and Disease

Many bacterial pathogens cause diseases of varying severity, and some cause no disease at all most of the time (e.g., [32], [33]), constituting a normal part of the body flora [2], [31]. Such observations raise the question of why bacteria cause disease and have led to the notion of the accidental pathogen [50], [51]. Virulence may be accounted for by differences between strains and by the expression of genes encoding toxins, adhesins, and drug resistance, often carried by mobile elements (e.g., [52]–[56]). With whole genome sequencing, populations of virulent and avirulent bacteria can be compared to help explain disease from mechanistic and phylogenetic standpoints. Koch's postulates might be revised [57] to cover the discovery of associations between disease and individual genes or alleles, a process that is likely to accelerate rapidly in the 21^st^ century. A fuller understanding of bacterial population structure may also help predict the effects of interventions such as vaccination.

Bacteria of the same species isolated from patients with different clinical presentations can be compared directly by whole genome sequencing. *Streptococcus pyogenes*, also known as Group A *Streptococcus*, causes benign pharyngitis and invasive disease including scarlet fever. A comparison of around 300 isolates from Ontario indicated that invasive bacteria do not form genetically distinct populations. Rather, closely related bacteria may be invasive or pharyngitis-associated, demonstrating that the ability to cause invasive disease is not restricted to specific strains [58], [59]. Evidence for adaptation in genes involved in capsule synthesis and virulence regulation supported a model in which mutation in vivo plays an important role in progression to invasive disease.

Reconstructing the relationships between strains with different clinical manifestations can reveal the evolutionary origins of disease. A study of disparate *Clostridium difficile* isolates including representative members of the hypervirulent lineages denoted ribotypes 017, 027, and 078 found they were descended from multiple ancestors in the species tree, consistent with a scenario in which virulence evolved several times during evolution [60]. Whole genome sequencing afforded improved clarity for reconstructing the genealogy of *Chlamydia trachomatis* and appeared to show that strains causing trachoma, an eye infection, were evolutionarily descended from an ancestor causing urogenital disease [61].

The iatrogenic effect of public health intervention on bacterial pathogen populations, such as the introduction of a novel vaccine or the withdrawal of an antibiotic from agricultural use, is of major importance. Vaccine escape in *Streptococcus pneumoniae* has been a concern since the introduction of the heptavalent conjugate polysaccharide vaccine PCV7 in 2000 as it protects against many, but not all, serotypes. Two genomic studies found evidence for capsular switching, in which hybrid strains normally covered by the vaccine but expressing non-vaccine serotypes arise through recombination [62], [63]. One such strain has quickly established in the United States, spreading westwards from New England [63].

## Real-Time Pathogen Genomics

The relentless demand for higher throughput, lower cost DNA sequencing has spurred dramatic advances in the capacity and rapidity of whole genome sequencing. Benchtop sequencers permit real-time applications of genomics by sequencing small batches of bacteria in a matter of hours. The outbreak of cholera on the island of Haiti in October 2010 provided an early example of the potential for real-time genomics [64]. In the wake of the devastating earthquakes of January 2010 that killed 230,000 people, two million residents were displaced from their homes. Cases were first reported on October 19. By July 2011, 419,511 cases and 5,968 deaths had been reported [65]. Initial investigation found many patients had drunk untreated river water. By November 1, 2010 culturing and PFGE confirmed the pathogen as *Vibrio cholerae* of probable South Asia origin [66]. First-round whole genome sequencing of Haitian isolates began on November 10 and completed within 2 days [64]. Genomic analysis showed they were essentially identical, but distinct from other cholera circulating in Latin America, instead resembling widely circulating Asia strains [64], a finding that was consistent with possible introduction by United Nations Peacekeeping troops dispatched from Nepal following the earthquakes [65].

Real-time genomics may prove particularly valuable in outbreaks involving newly emerged strains. However, processing genomic data in real time poses considerable analytical challenges. In the May 2011 outbreak of *Escherichia coli* in Germany, a novel crowd-sourcing experiment was trialed that foretells of the potential of real-time genomics to radically alter the way outbreaks are investigated [67]. The large outbreak was unusual in several aspects: high incidence in adults, greatly increased incidence of hemolytic-uremic syndrome, a preponderance of female patients, and a rare, Shiga toxin-producing serotype not previously linked to outbreaks [67], [68]. A first draft of the genome of an isolate sampled on May 17 was completed within 3 days, then released into the public domain, eliciting curiosity-driven analysis by scientists on four continents [67]. Within a week two dozen reports had been filed on a dedicated open-source wiki. Analysis of this and other strains concluded that the outbreak was caused by the acquisition of a Shiga toxin-encoding prophage and a plasmid bearing an extended-spectrum beta-lactamase gene by an ancestral enteroaggregative strain [67], [68]. The striking virulence of the hybrid may be connected to the atypical presence of three SPATE genes, which are implicated in mucosal damage and intestinal colonization [68].

Two studies from hospitals in the United Kingdom have demonstrated the practical advantages of real-time whole genome sequencing as part of routine outbreak investigation and surveillance. Focusing on the most serious health-care-associated pathogens, *C. difficile* [69] and *S. aureus* [69], [70], bacterial samples were isolated from suspected outbreaks in four hospitals. The genomes were sequenced and analyzed within 5 working days of culture, confirming the suspected outbreaks of MRSA (methicillin-resistant *S. aureus*), but demonstrating that the epidemiologically linked cases of *C. difficile* infection were in fact genetically distinct. Characterization of the repertoire of resistance and toxin genes provided further information relevant to patient management.

## Summary and Perspectives

In the future, population genomics will be central to an improved understanding of the epidemiology, etiology, and evolution of bacterial infectious diseases. However, there are obstacles yet to overcome. Pilot studies have demonstrated the potential genomics has for epidemiological investigation [40], [41], [64], [67]–[70], but creative solutions to the problem of integrating complex epidemiological and genomic data are now required. Currently, genome sequencing relies on culture to yield sufficient bacterial DNA and new technologies are needed to overcome this dependency. If the cost of DNA library preparation can be substantially reduced, genomics will come within reach of public health authorities as a tool for routine surveillance. These and other future challenges are discussed in Box 1.

**Box 1. Future Challenges for Pathogen Whole Genome Sequencing**

High-throughput whole genome sequencing has been demonstrated to be a practical tool for epidemiological and evolutionary investigation of bacterial pathogens, yet the current technology has certain limitations. The challenge for future advances in sequencing technology is to overcome these problems.

**Culture.** Reliably sequencing the genomes of individual bacteria requires culture to obtain sufficient quantities of concentrated DNA. This takes time and effort, restricts the approach to culturable organisms, and may introduce artifacts such as in vitro mutation and laboratory cross-contamination. Direct sequencing without culture (e.g., [81]) may in the future relinquish this dependency on culture, but metagenomics approaches present additional challenges for bioinformatics and sequence analysis (see, e.g., [82]).

**Library preparation.** Exponential increases in the capacity of high-throughput sequencers show no sign of abating. In principle, this should allow the cost of bacterial whole genome sequencing to continue to fall. However, the price per genome also depends on the cost of DNA library preparation, comprising both consumables and labor. Advances in automation and throughput will be required to prevent library preparation becoming a bottleneck, and to reduce the cost sufficiently that bacterial genome sequencing becomes affordable for routine surveillance.

**Bioinformatics.** The development of bespoke bioinformatics pipelines for bacterial whole genome sequencing represents a considerable investment and a complex set of choices from among the many computational methods on offer. Some degree of normalization is required to ease the burden on users of whole genome sequencing, for example hospital microbiology laboratories, and to promote standardized and replicable workflows.

**Platforms.** High-throughput sequencing technologies yield large quantities of short read sequences but with substantially elevated error rates, compared to conventional capillary sequencing. The details of sequence length and error profile differ in important ways between platforms. In consequence, different results may be obtained when the same sample is sequenced on different platforms. Improved understanding of the error profiles of different architectures combined with efforts towards quantifying uncertainty in the DNA sequences generated will help minimize discrepancies of this kind.

**Genome assembly.** De novo assembly is used to join together the short reads of DNA generated by the sequencing machines into longer genome fragments, known as contigs. The ultimate goal is to join all the fragments into a single contig representing the whole bacterial chromosome, known as a closed genome. However, variation in the number of reads sequenced from each part of the genome (the depth of coverage), and the existence of repetitive regions, conspire to prevent this. With longer reads, it should be possible to overcome these problems.

**Public databases.** To accelerate the pace of discovery and assist collaboration between laboratories, well-organized publicly available databases are required from which bacterial genomes are readily downloaded in convenient formats. Raw data are currently available in short read archives (e.g., http://www.ncbi.nlm.nih.gov/sra and http://www.ebi.ac.uk/ena), but with standardization of bioinformatics processing it should become possible to provide pre-processed data which would dramatically reduce the workload for database users.

Improved understanding of disease etiology helps to direct research into therapies. Genomics is a promising tool for investigating the differences between invasive and non-invasive bacteria at the population and within-host levels [27], [59]–[61]. Tools from human genetics may help in this endeavor. Even so, investigations into bacterial population structure are required to assess the feasibility of genome-wide association studies [71]. Understanding the architecture of traits such as virulence would benefit from the development of high-throughput phenotyping assays. RNA sequencing is one such candidate [72], but differences in gene expression in culture and in vivo are a potential impediment to progress.

Population genomics also promises to improve our understanding of bacterial pathogen evolution. The resolution of whole genome sequencing allows precise calibration of evolutionary rates from longitudinal samples within populations and individual hosts [16]–[18], [28], [35], [40], [47]. This permits the origin of new species to be dated, but the discrepancy between short- and long-term rates requires further explanation [23], [30]. Investigating within-host dynamics will help identify the evolutionary mechanisms involved in disease progression [27], [35]. Sequencing populations of pathogens will reveal the prevalence of virulence factors and drug resistance, and the role of mobile elements in their spread [44]. Ultimately, however, we must pinpoint the evolutionary advantages that bacteria gain from inflicting illnesses if we are to fully understand the causes of bacterial disease.
